# Multichemical Exposure Assessment Using Silicone Wristbands in Central American Adolescents

**DOI:** 10.1007/s12403-026-00759-y

**Published:** 2026-03-09

**Authors:** Yirong Yuan, Samantha M. Hall, Juan José Amador Velázquez, Damaris López Pilarte, Selene Vences Brown, Magaly Amador Sánchez, Juan Amador Sánchez, Birgit Claus Henn, Madeleine K. Scammell, David J. Friedman, Daniel R. Brooks, Jessica H. Leibler

**Affiliations:** 1 Department of Environmental Health, Boston University School of Public Health, 715 Albany St, Boston, MA 02118 USA; 2 Centro Médico del Pacífico (CENMED), Masaya, Nicaragua; 3 Division of Nephrology, Beth Israel Deaconess Medical Center, Harvard Medical School, Boston, MA USA; 4 Department of Epidemiology, Boston University School of Public Health, Boston, MA USA

**Keywords:** Chemical exposure, Chemical mixture, Silicone wristband, CKDu, Mesoamerican nephropathy, Central America

## Abstract

Chemical exposures are poorly characterized in Central America, where chronic kidney disease of uncertain etiology (CKDu) is a leading cause of death. Most research evaluates occupational exposures in adults, yet early-life exposures remain understudied. We used silicone wristband passive samplers to characterize exposure to over 1,500 chemicals among Nicaraguan adolescents (*n* = 80) residing in CKD high-risk communities but not engaged in agricultural work. We identified exposure profiles using k-means clustering and used LASSO logistic regression to identify predictors of low estimated glomerular filtration rate (eGFR). SHAP (SHapley Additive Explanations) values quantified individual chemical contributions to eGFR in a machine learning model. Of 130 detected chemicals, 21 (16.2%) were pesticides. Pyrethroids were detected on 93.8% of wristbands, most frequently cypermethrin-2 (88.8%) and ethofenprox (60.0%). Phthalates and musks, including bis(2-ethylhexyl) phthalate (DEHP, 98.8%), di-n-butyl phthalate (DnBP, 97.5%), and galaxolide (100%), were near ubiquitous. Esters included benzyl salicylate (97.5%) and ethylene brassylate (93.8%). Organophosphate triphenyl phosphate (TPP, 97.5%) and polyaromatic hydrocarbons (PAHs) pyrene (92.5%) and 2-methylphenanthrene (63.8%) reflected mixed consumer and environmental sources. K-means clustering identified four exposure profiles: two larger clusters reflecting background agrochemical exposures and a low-exposure group, and two smaller clusters dominated by household and personal care products. No individual chemicals were significantly associated with eGFR. SHAP analysis identified phthalates, esters, organophosphates, and phenols as the most influential predictors of kidney function. Adolescents in this CKDu-endemic region experience heterogeneous chemical exposures. Frequently detected compounds with nephrotoxic potential, including pyrethroids, PAHs, phenols, and phthalates, warrant targeted investigation.

## Introduction

Chronic kidney disease of uncertain or non-traditional origin (CKDu or CKDnt) is a global epidemic and is often termed Mesoamerican Nephropathy (MeN) in Central America (Johnson et al. 2019). The disease typically presents in early adulthood without comorbid conditions such as hypertension, diabetes or obesity and progresses rapidly (Lusco et al. 2017). Exposure to heat stress in tropical climate, typically through intense, outdoor physical labor, is an established risk factor for the disease, although the disease is widely considered to be multifactorial and the full causal structure remains unclear (Brooks et al. 2012; Garcia-Trabanino et al. 2015; Glaser et al. 2016; Gallo et al. 2022).

MeN is particularly prevalent among male agricultural workers, and occupational pesticide exposure has long been a leading causal hypothesis (Valcke et al. 2017). Many agrochemicals are known or suspected nephrotoxicants, including paraquat, 2,4-D, and organophosphates (Jayasumana et al. 2014; Scammell et al. 2019; Wan et al. 2021; Jacobson et al. 2021). However, evidence to date has not depicted a clear association between exposure to pesticides or herbicides used in Central America and MeN. Furthermore, there is limited exposure information among young and non-working populations in this region that inform an understanding of susceptibility.

Childhood and adolescence pose important developmental windows of vulnerability in relation to chronic diseases of adulthood (Chevalier 2019). Our previous work has demonstrated that kidney injury and increased prevalence of poor kidney health is observed among Central American adolescents prior to working life in MeN-affected communities, indicating that pre-occupational factors are relevant in disease etiology (Leibler et al. 2020; Hall et al. 2023). The young age of onset of MeN, often in the 20s and 30s, also suggests that early life exposures are important in disease initiation or progression, but such factors have received limited scrutiny.

Silicone wristband passive samplers can assess individual-level chemical exposure to a broad range of compounds (O’Connell et al. 2014). These wristbands are easy to deploy in field observational studies and can provide nuanced detection and quantification for > 1500 chemicals (Dixon et al. 2019). Exposure data from silicone wristbands is correlated with biomarker data, indicating that the wristbands serve as a non-invasive proxy for internal dose (Hammel et al. 2018; Levasseur et al. 2024). The wristbands have notable value in identifying unrecognized exposures of relevance where a priori, targeted hypotheses have failed to do so, such as in the context of MeN. We deployed silicone wristbands among Nicaraguan youth at risk of CKDu to assess exposure to a broad range of chemicals, describe potential sources, and consider associations with kidney function.

## Methods

### Recruitment and Study Design

Participants for the silicone wristband sub-study were recruited from a prospective cohort of adolescents and young adults in Nicaragua. Approximately 600 participants had annual questionnaire and biospecimen collection (blood and urine) from 2022 to 2024. In fall 2023, 80 youth (40 males and 40 females) were selected to participate in the wristband sub-study from two high-risk MeN regions that differed in industrial presence. Given the exploratory aims of this analysis and the high-dimensional exposure setting, formal a priori power calculations were not performed. Chinandega is the leading sugarcane production region in Nicaragua and considered the epicenter of the MeN epidemic in that country (Brooks et al. 2012). As a non-agricultural referent, we recruited in León, where there are also high rates of MeN but where the predominant industries are mining and brick-making.(Gallo-Ruiz et al. 2019) We recruited participants in a 2:1 ratio between the regions so as to oversample participants with presumed agrochemical exposure, and with even sex and age distribution by region. Eligibility criteria were having never worked in agriculture; full-time or part-time student status; and residing in one of the study communities. Participants meeting these criteria were recruited on the basis of age so as to identify exposures among younger participants.

### Wristband Deployment

Silicone wristbands were obtained from MyExposome (Corvalis, OR) and decontaminated prior to deployment by heating to 280 C (O’Connell et al. 2014). Participants wore the wristband 24 h/day for 7 days during all activities, and sampling took place during the annual follow-up period (September to November 2023). The wristband was returned to study staff in a disinfected plastic bag at the end of the week and were stored in ambient conditions until shipment. All participants provided informed consent, and all study protocols were approved by the Boston University Medical Campus IRB and the Nicaraguan Ministry of Health (MINSA).

### Laboratory Analysis

MyExposome conducted the chemical analysis, screening for a total of 1,530 chemicals. Wristbands were rinsed with 18 MΩ·cm ultrapure water, followed by a rinse with isopropanol and storage at − 20 °C in individual amber glass jars until extraction. Wristbands were subjected to sequential extraction using two 50 mL volumes of ethyl acetate. Extracts were combined, and the sample volume was reduced to 1 mL via evaporation. Concentrated samples were stored at 4 °C before undergoing solid-phase extraction with C18 columns and acetonitrile. The purified extracts were solvent-exchanged into iso-octane and stored again at 4 °C until analysis.

C = Gas chromatography (6890 N Gas Chromatograph, Agilent, Santa Clara, CA, USA) and mass spectrometry (5975B Mass Selective Detector) were employed to determine chemical composition, utilizing Automated Mass Spectral Deconvolution and Identification System (AMDIS) software. The detection limits, reporting thresholds, and analytical performance parameters followed established protocols (O’Connell et al. 2014). The amount of each detected chemical was expressed in nanograms of chemical per gram of wristband. If a specific chemical was not detected, a zero value was assigned. Quality control measures included blank unworn wristbands, solvent extraction blanks, instrument blanks, and continuing calibration verification samples.

### Statistical Analysis

Descriptive statistics were evaluated for all wristbands by compound, region, and sex. Summary statistics, comparison tests and Spearman’s correlations were assessed for every compound detected in more than 50% of study participants, and these chemicals were included in subsequent analyses. For each chemical, we approximated a detection limit (LOD) as that chemical’s smallest positive value, multiplied by 10^− 6^, with a hard floor of 10^− 12^ (Hornung and Reed 1990). In the primary analysis, concentrations of chemicals that were less than or equal to zero were imputed as LOD divided by the square root of two. A sensitivity analysis was conducted using the LOD value directly. Concentrations above the LOD were maintained. Dimensionality was reduced by aggregating individual chemicals into nine chemical classes, which were used as the exposure variables in all regression analyses: alkaloid, esters, organophosphate, PAHs, other aromatics, phenols, phthalates, pyrethroids, and synthetic musks. Within each chemical, we winsorized values to the 5th–95th percentiles (capped, not excluded) and then min–max scaled them to 0–1. Group‑level exposure for each participant was the sum of the scaled chemicals in that group, analyzed on the log scale. Single chemical models used the log of the imputed concentration or log of the concentration $>$LOD.

### Exposure Profile Clusters

K-means clustering was used to identify common multi-chemical signatures and explore potential shared exposure sources. Clusters including ≥ 5 individuals were visualized and depicted. We selected the number of clusters that captured the majority of variance while minimizing over-partitioning of the data.

### Associations with Kidney Health

GFR was estimated using the serum creatinine-based European Kidney Function Consortium (EKFC) equation which uses sex- and age-specific constants to account for creatinine fluctuations during adolescence and early adulthood (Pottel et al. 2021). We calculated correlations between eGFR and scaled chemical group exposure concentrations. We used LASSO regression adjusted for age and sex to reduce multidimensionality and assess exploratory chemical associations with low eGFR (< 90 mL/min/1.73m^2^) (KDIGO 2024), and we conducted sensitivity analyses adjusting for residential region.

We applied SHapley Additive exPlanations (SHAP) values to interpret how individual chemical groups contributed to predictions of reduced kidney function (eGFR < 90 ml/min/1.73m^2^) (Fu et al. 2024). SHAP values quantify the marginal effect of each feature on the model’s output for each participant, indicating whether a given chemical increased or decreased the predicted risk of low eGFR. This approach facilitates interpretation of complex exposure-health relationships in high-dimensional environmental data and smaller samples. SHAP values were calculated for the XGBoost model (TreeSHAP) used to evaluate associations between chemical group exposures and low eGFR, providing model-based estimates of the relative contribution of each exposure group to the predicted probability of reduced kidney function.

## Results

### Study Participants

Eighty individuals (*n* = 80) enrolled in this study, 40 females and 40 males. Among participants, 56 (70%) resided in Chinandega and 24 (30%) resided in León. All participants had resided in the study region for at least five years. Mean age of participants was 18.3 years (range: 14–26) and 28.8% (*n* = 23) were full-time students and had never worked. Mean eGFR among participants was 98.1 mL/min/1.73m^2^ (SD: 15.9) which did not differ significantly by sex or from the full cohort. Prevalence of eGFR < 90 mL/min/1.73m^2^ was 15.0% (*n* = 12), including six males and six females.

### Descriptive Statistics

Mean eGFR was 102.2 (SD: 12.1). A total of 130 chemicals were identified on at least one wristband (Supplement Table 2). The median number of chemicals detected on a single wristband was 24 (IQR: 8, min: 9, max: 53). Across 80 wristbands, a broad spectrum of semi-volatile organic compounds was detected, reflecting overlapping exposures from household, personal care, agricultural, and combustion sources. Phthalates were nearly ubiquitous, with bis(2-ethylhexyl) phthalate (98.8%), diisobutyl phthalate (98.8%), di-n-butyl phthalate (97.5%), and diethyl phthalate (97.5%) each detected in nearly all participants, indicating widespread contact with plasticizers and personal care product ingredients. Synthetic musks were also highly prevalent, including galaxolide (100%), tonalide (92.5%), and cashmeran (22.5%), consistent with frequent use of fragranced consumer products. The organophosphate ester triphenyl phosphate (TPP) (97.5%) and tris(1-chloro-2-propyl) phosphate (TCPP) (6.3%) were also detected, likely reflecting indoor exposures to flame retardants and plastic additives.

Esters were recovered on a majority of wristbands, with benzyl salicylate (97.5%), ethylene brassylate (93.8%), and benzyl benzoate (36.3%) frequently detected, indicating fragrance or fixative use in consumer products. Among aromatic compounds, Lilial (78.8%), 2,4-di-tert-butylphenol (70.0%), benzophenone (45.0%), coumarin (27.5%), and biphenyl (26.3%) were recovered, reflecting exposures to fragrance additives, antioxidants, and industrial intermediates. Aldehydes such as amyl cinnamal (33.8%) and citral A (26.3%), and the terpenoid β-ionone (31.3%) indicate exposure to fragrance components. Phenols exposure included butylated hydroxytoluene (18.8%) and 4-tert-butylphenol (10.0%) reflect exposure to antioxidant additives in plastics and food-contact materials.

Chemicals of agricultural and combustion origin were also observed in high prevalence. Pyrethroid insecticides were detected in a substantial proportion of participants, including cypermethrin-2 (88.8%), ethofenprox (60.0%), permethrin (35.0%), and deltamethrin (18.8%), consistent with exposure to insecticides used in sugarcane cultivation and domestic pest control. Polycyclic aromatic hydrocarbons (PAHs) were among the most chemically diverse classes, with high-prevalence compounds including pyrene (92.5%), 2-methylphenanthrene (63.8%), and anthracene (57.5%), and lower-prevalence species such as 1-methylphenanthrene (38.8%), fluoranthene (21.3%), and benz[a]anthracene (25.0%). These findings are consistent with exposure to combustion by-products from sugarcane burning, diesel exhaust, and biomass fuel use.

To improve interpretability and statistical robustness, subsequent analyses were restricted to compounds detected in at least half of all wristbands (≥ 50%). This subset comprised 19 chemicals spanning eight chemical classes (Table 1). Phthalates were most represented (*n* = 6), followed by polycyclic aromatic hydrocarbons (*n* = 3), synthetic musks (*n* = 2), esters (*n* = 2), and pyrethroids (*n* = 2). Single chemicals were included for organophosphate esters, phenols, alkaloids, and other aromatics. These frequently detected chemicals provide a focused set of exposures for subsequent clustering and association analyses.

**Table 1:** Chemicals recovered on ≥ 50% of silicone wristbands among Nicaraguan adolescents and young adults, ages 13–23 years (*n* = 80)

Chemical	Chemical Group	% Detected	Median detected^1^ concentration (IQR) (ng/g)	All-sample (imputed) median (IQR) (ng/g)
Galaxolide	Synthetic Musks	100	26,000 (13,000, 46,000)	26,000 (13,000, 46,000)
Bis(2-ethylhexyl)phthalate	Phthalate	98.8	2,200 (1,100, 6,750)	2,150 (1,100, 6,625)
Diisobutyl phthalate	Phthalate	98.8	16,000 (7,500, 30,500)	15,000 (7,500, 30,250)
Benzyl salicylate	Esters	97.5	11,500 (5,425, 23,750)	10,500 (5,250, 23,250)
Di-n-butyl phthalate	Phthalate	97.5	36,500 (22,000, 59,000)	36,000 (19,750, 59,000)
Diethyl phthalate	Phthalate	97.5	1,700 (660, 5,175)	1,650 (580, 5,125)
TPP	Organophosphate	97.5	1,400 (552, 2,575)	1,350 (532, 2,525)
Ethylene brassylate	Esters	93.8	16,000 (4,900, 44,000)	13,500 (4,550, 39,750)
Pyrene	PAHs	92.5	210 (120, 288)	210 (110, 280)
Tonalide	Synthetic Musks	92.5	665 (355, 3,300)	635 (208, 3,225)
Cypermethrin-2	Pyrethroids	88.8	1,300 (655, 2,650)	1,100 (512, 2,225)
Lilial	Other Aromatics	78.8	830 (445, 1,600)	575 (220, 1,200)
2,4-Di-tert-butylphenol	Phenols	70	445 (230, 860)	270 (0, 560)
Butyl benzyl phthalate	Phthalate	70	630 (425, 1,400)	440 (0, 892)
2-Methylphenanthrene	PAHs	63.8	120 (63, 140)	58 (0, 122)
Ethofenprox	Pyrethroids	60	2,200 (960, 7,125)	825 (0, 4,175)
Caffeine	Alkaloid	58.8	3,400 (1,600, 5,250)	1,015 (0, 4,225)
Anthracene	PAHs	57.5	155 (90, 205)	73 (0, 170)
Di-n-nonyl phthalate	Phthalate	51.2	1,800 (1,300, 2,800)	235 (0, 1,875)

^1^% Detected” was computed before imputation. “All-sample (imputed)” summarizes concentrations after replacing non-detects with a chemical-specific near-zero value (tiny / √2) used for modeling. Values shown as 0 indicate concentrations < 1 ng/g after rounding and do not represent true zeros; all non-detects were imputed with small positive near-zero values for modeling

### Exposure Correlations

We observed moderate, statistically significant positive pairwise correlation between chemical groups, suggestive of shared exposure sources, as follows: synthetic musks and esters (*r* = 0.62); pyrethroids and PAHs (*r* = 0.50); synthetic musks and other aromatics (*r* = 0.49); phthalates and phenols (*r* = 0.47). Pyrethroids were also moderately correlated with phenols (*r* = 0.44), indicating potential co-occurrence or shared exposure sources. Compared with León, participants in Chinandega had higher summed group-level exposures for pyrethroids (Wilcoxon *p* = 0.0027; FDR q = 0.024) and PAHs (*p* = 0.0085; FDR q = 0.038).

### Clustering

**Fig. 1:**
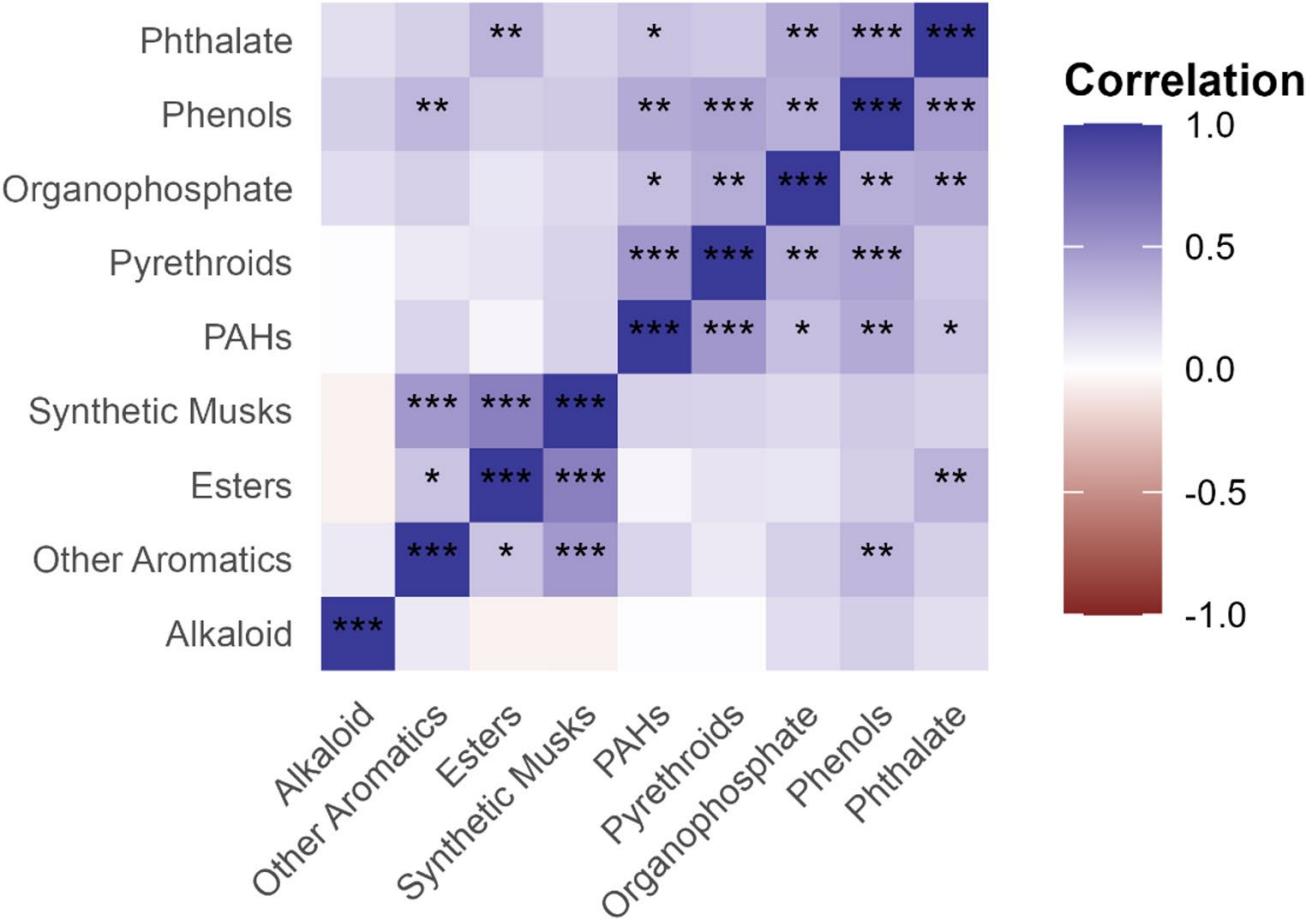
Pairwise spearman correlation among chemical groups. The heatmap shows pairwise Spearman correlation coefficients (r) between group-level exposure indices (each index was winsorized within-analyte to the 5th–95th percentile, rescaled to 0–1, summed within group, and log-transformed). Cells are colored by r (diverging scale, − 1 to + 1). q-values were controlled using the Benjamini–Hochberg false discovery rate (FDR) method; significance is indicated by *, **, *** for q < 0.05, q < 0.01, and q < 0.001, respectively

K-means clustering identified four primary exposure profiles (Fig. 1) consistent with heterogenous exposures but dominated by two profiles: background agrochemical exposures and a generalized low exposure profile. Cluster 1 (*n* = 27; “background agrochemical exposures”) indicated modest co-elevations of pyrethroids, PAHs, and phthalates, representing a pattern consistent with mixed ambient agrochemical exposures. Cluster 2 (*n* = 30; “low exposure”) depicted lower than mean exposure concentrations across all chemical groups, with PAHs, phthalates, pyrethroids, and phenols concentrations below the mean. Two smaller clusters were driven by household and personal care products. Cluster 3 (*n* = 11; “household products”) was characterized by elevated concentrations of TPP, Lilial, and 2,4-di-tert-butylphenol, indicating predominant exposure to household or indoor consumer-products, including flame retardants, fragrance additives, and antioxidant preservatives. Cluster 4 (*n* = 12; “personal care products”) included elevated concentrations of esters, synthetic musks, and phthalates, and moderate levels of PAHs and phenols. This cluster reflects a personal-care and consumer-product exposure profile, characterized by high levels of fragrance additives, plasticizers, and semi-volatile organic compounds typical of cosmetics, detergents, and household products. Cluster membership did not vary significantly based on sex or region.

### Chemical Exposures and eGFR

**Fig. 2:**
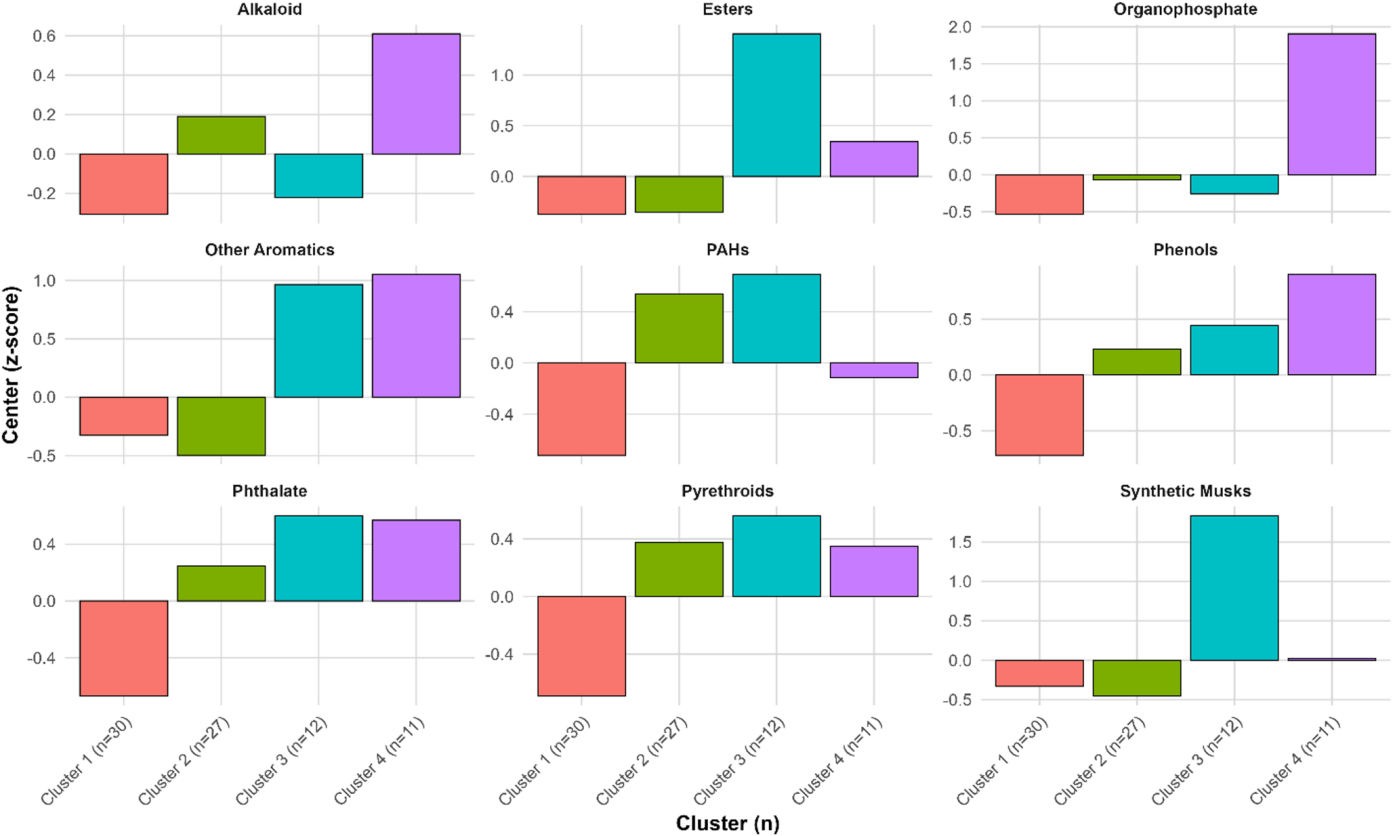
Chemical exposure clusters among Nicaraguan adolescents and young adults (*n* = 80). K-means clustering identified four exposure profiles: (Cluster 1, red) *Background agrochemical exposures* (*n* = 27), with co-elevations of pyrethroids, PAHs, and phthalates; (Cluster 2, green) *Low exposure* (*n* = 30), with concentrations below the mean across all chemical groups; (Cluster 3, teal) *Household products* (*n* = 11), enriched in TPP, Lilial, and 2,4-di-tert-butylphenol; and (Cluster 4, purple) *Personal care products* (*n* = 12), characterized by elevated esters, musks, and phthalates. Cluster membership did not differ by sex or region

Spearman correlations between continuous eGFR and group‑level exposures were uniformly small (|r| ≤ 0.13) and none were significant after FDR correction. In sex‑ and age‑adjusted LASSO using the 9 chemical exposure groups and a dichotomous eGFR outcome, no group was retained at the 1‑SE penalty. In a per‑chemical LASSO restricted to the 19 analytes with ≥ 50% detection, only benzyl salicylate was selected at the 1‑SE penalty, but post‑LASSO single‑analyte logistic models (sex‑ and age‑adjusted) showed no association with low eGFR (< 90 ml/min/1.73m^2^) after FDR correction. Model performance was evaluated using 5-fold stratified cross-validated metrics and was modest and comparable to covariate-only baselines, consistent with the exploratory nature of analysis. K-means clusters did not differ significantly by eGFR in ANOVA analyses. Results were consistent in sensitivity analyses with additional adjustment for residential region (Fig. 2).

**Fig. 3:**
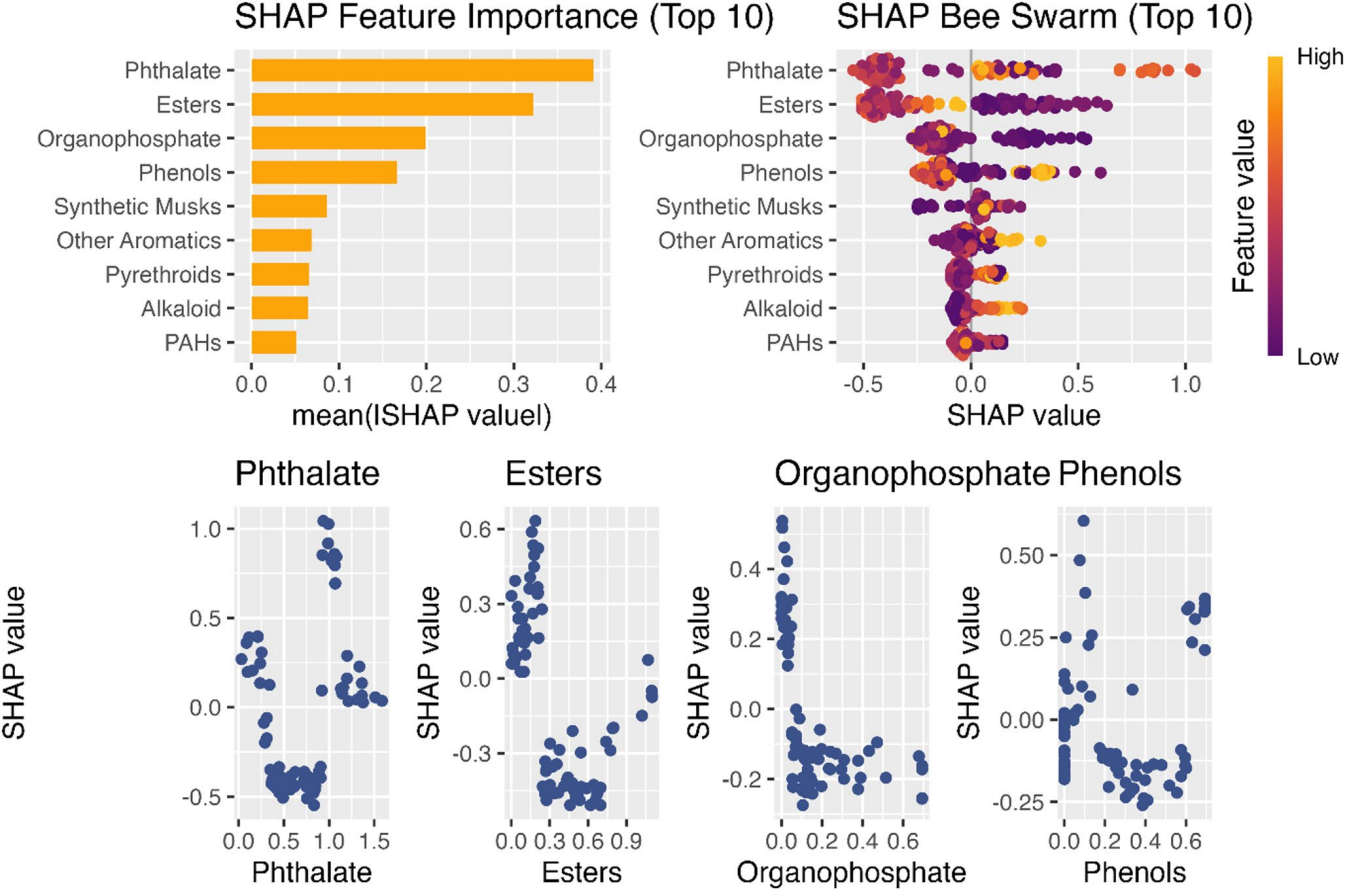
SHAP feature importance and dependence plots (*n* = 80). SHAP (SHapley Additive Explanations) results from the XGBoost model predicting eGFR status. Top left: Feature importance ranked by mean absolute SHAP values. Top right: Bee swarm plot showing the distribution and direction of feature effects (color = exposure level). Bottom: Dependence plots for the top four features

The SHAP feature importance plot (Fig. 3, top left) indicated that the strongest contributing features to eGFR were phthalates (mean |SHAP| ≈ 0.40 ), esters (≈ 0.32), organophosphates (≈ 0.20), phenols (≈ 0.17), and synthetic musks (≈ 0.09). Moderately important features included pyrethroids, alkaloids, and PAHs. The SHAP bee swarm plot (Fig. 3, top right) visualizes the influence of each chemical group on predicting reduced kidney function (eGFR < 90 ml/min/1.73m^2^). Each dot represents a participant, with color indicating exposure level (purple = low, yellow = high) and horizontal position reflecting impact on the model’s prediction. The bee‑swarm plot indicates that most observations had small SHAP values, but a subset of participants with higher phthalate or phenol levels exhibited positive SHAP values, aligning with a higher model‑predicted risk of low eGFR. In contrast, esters and organophosphates generally showed negative SHAP values at elevated concentrations, implying an inverse relationship in the tree‑based model.

The dependence plots (Fig. 3, bottom) depict variation in SHAP values by exposure concentration, though no strong monotonic pattern was observed. Phthalates, esters, organophosphate (TPP) and phenols were most strongly aligned with lower predicted eGFR. Despite these trends, relationships were mixed for all chemical classes, with inverse or potentially non-linear relationships observed in some individuals. Overall, the model indicates that consumer product and household chemicals were the primary exposures associated with reduced kidney function.

## Discussion

This analysis describes chemical exposure profiles among adolescents and young adults living in a Central American region with high CKDu prevalence, providing novel data on agrochemicals, household products, and personal care exposures. Distinct mixtures were identified, including an environmental agrochemical profile with pyrethroids and PAHs, and clusters dominated by household and consumer products such as phthalates, esters, and synthetic musks. No clear associations with kidney function were observed. Overall, these findings highlight heterogeneous, low-level exposures spanning agricultural and household environments, including some that may contribute to chronic kidney disease risk over time.

Galaxolide and phthalates DEHP and DIBP were the most prevalent recovered exposures, detected in nearly all participants. Galaxolide is a synthetic musk widely used as a fragrance in personal care products, detergents, and air fresheners. It is a lipophilic compound with environmental persistence and is widely detected in the environment and human studies (Hutter et al. 2005). Galaxolide may exhibit endocrine-disrupting activity, though evidence linking it to kidney toxicity is limited (Xi et al. 2025). DEHP and DIBP are widely used plasticizers with documented nephrotoxic effects in animal studies (Payne-Sturges et al. 2023). In humans, urinary metabolites of DEHP and DIBP has been associated with reduced eGFR and biomarkers of kidney injury (Chang et al. 2021; Li et al. 2025). Possible synergistic effects on kidney health merit further investigation.

An agrochemical exposure cluster, including approximately 25% of participants, reflected compounds linked to regional agricultural production, notably pyrethroids (cypermethrin-2 and etofenprox) and polycyclic aromatic hydrocarbons (PAHs; anthracene, pyrene, and 2-methylphenanthrene). Pyrethroid insecticides are widely used in sugarcane cultivation, with experimental and epidemiologic evidence suggesting potential renal toxicity in acute or high-level exposure scenarios. PAHs are generated through incomplete combustion of organic materials and fuels and may arise from the burning of sugarcane fields during harvest or from diesel exhaust produced by farm machinery. The study was conducted during the pre-harvest period (November through January), when burning is routinely used to facilitate cutting and transport, and the concurrent elevation of pyrethroids and PAHs likely reflects co-exposure to these industrial agricultural activities. Additional sources, such as wood burning in brick kilns near León, also contribute to regional PAH emissions, suggesting that multiple agricultural and industrial processes likely influence the air quality and background exposure burden in surrounding communities.

Both pyrethroid and PAH exposure are linked to oxidative stress and endocrine disruption (Kim et al. 2013; Ravula and Yenugu 2021). Co-exposure to these chemical classes may result in synergistic or cumulative nephrotoxicity, particularly among children and adolescents, whose kidneys are still developing and may have reduced capacity to detoxify environmental contaminants (Gomez et al. 1999). This underscores the relevance of evaluating chemical mixtures in relation to kidney health outcomes. Some of the pyrethroid compounds assessed here were personal care products for scabies and lice treatments, while others were used in industrial agriculture. The size of our sample limits our ability to distill the importance of individual compounds in relation to eGFR or exposure clusters.

Male participants had significantly higher concentrations of esters, terpenoids, alcohols, and phenols, compared to females. These differences may reflect gendered patterns in personal products or occupational activities, although these sex-specific exposures were not associated with low eGFR. We observed correlations between musks and esters, compounds used as fragrances, potentially suggesting that individuals using fragranced products may use multiple scented products (e.g. perfumes, laundry scents, hair products).

SHAP analysis identified esters (benzyl salicylate, ethylene brassylate), organophosphate esters (triphenyl phosphate), and phenolic compounds (2,4-di-tert-butylphenol) as the main contributors to variation in eGFR, although effect sizes were small and overall model performance was modest. These exposure groups reflect fragrance additives, flame retardants, and antioxidant preservatives, respectively, chemicals commonly found in indoor environments and personal care products. The SHAP dependence plots suggested moderate, and potentially nonlinear or threshold-dependent relationships with eGFR, broadly consistent with endocrine or oxidative stress related pathways. However, the lack of strong, consistent, or directionally stable associations across statistical methods indicates that within this study, none of the measured chemical classes were strongly associated with kidney function.

Study limitations include a non-occupational population, which may limit the ability to identify proximate risk factors for CKDu, a disease prevalent among agricultural workers. Our intention was to characterize community-level exposures to depict non-occupational chemical profiles; occupational studies are better suited to quantify exposures associated with agricultural work. Relatively small sample size reduced power to detect subtle exposure-response relationships. The study period (September–November) may not have captured periods of peak agrochemical use, as pesticide and insecticide applications in sugarcane typically occur earlier in the year (February–June). It is possible that temporal variation during our study period affected results. While all participants in this substudy reported using municipal water sources, analysis of drinking water sources would support an understanding of exposure sources. The one-week wristband wear period reflects short-term exposure and may not represent seasonal or chronic exposure profiles. The analytic approach used here could not recover glyphosate, which is widely applied in regional sugarcane production and a hypothesized but unconfirmed CKDu risk factor. Silicone wristbands capture semi-volatile organic compounds but not highly polar or non-volatile species, potentially underrepresenting certain pesticide classes and metals. The cross-sectional eGFR endpoint used here may not fully reflect early renal injury and we are unable to discern more sensitive kidney injury or normal variation. As LASSO models were used for primarily for variable screening, we did not evaluate or interpret predictive performance metrics.

SHAP modeling can be unstable in small samples with correlated features, and reflects contributions to model predictions, rather than biological importance. Consistent with the exploratory aims and limited sample size, discrimination was modest and comparable to covariate-only baselines. Baseline covariates offered limited predictive signal in this cohort (e.g., low eGFR cases were evenly distributed by sex and the age range was narrow), which likely contributed to minimal separation between null and baseline models. These models identify variable importance but do not imply causality. Likewise, k-means clustering with a small sample may produce less stable assignments and clustering could be considered exploratory. Although penalized regression and exposure aggregation reduce dimensionality, residual overfitting remains possible given the sample size. Chemical classes were moderately correlated, reflecting shared exposure sources and co-occurrence of chemicals in personal environments. As a result, estimated associations for individual classes should be interpreted as indicators of broader exposure patterns rather than isolated effects of single chemical groups. More complex ensemble or multi-algorithm machine learning approaches may offer advantages in larger datasets; however, given the modest sample size, we prioritized model parsimony and interpretability to reduce overfitting risk. Future analyses in larger cohorts are valuable to evaluate selection stability and to apply more flexible mixture modeling approaches.

Despite these limitations, this study represents among the first efforts to characterize complex chemical mixtures with relevance to CKDu using silicone wristbands. The study design integrates high-resolution, untargeted exposure assessment with machine learning and clustering approaches to evaluate early-life environmental determinants of kidney health. Our findings inform priorities for targeted biomonitoring and mechanistic research in CKDu-endemic regions.

## Conclusion

In cross-sectional analysis of Nicaraguan adolescents, four distinct exposure profiles were identified, representing household, agricultural, and personal care product mixtures. Phthalates, esters, and synthetic musks dominated overall exposures, while pyrethroids and PAHs characterized an agricultural exposure subgroup consistent with environmental exposure to sugarcane production. We identified four distinct exposure profiles reflecting mixtures of household, agricultural, and personal care product chemicals. Although no clear associations with eGFR were observed, these findings highlight early-life exposure to complex chemical mixtures in a CKDu-endemic region. Despite small sample size, frequently detected compounds with known or suspected nephrotoxic potential, including pyrethroids, PAHs, phenols, and phthalates, suggest hypotheses for future prospective research and warrant targeted evaluation.

## Data Availability

The dataset used in this analysis is publicly available at: https://github.com/yiryuan/CKDu_Wristbands2025.
